# Forecasting Nonlinear Systems with LSTM: Analysis and Comparison with EKF

**DOI:** 10.3390/s21051805

**Published:** 2021-03-05

**Authors:** Juan Pedro Llerena Caña, Jesús García Herrero, José Manuel Molina López

**Affiliations:** Applied Artificial Intelligence Group (GIAA), Carlos III University of Madrid, 28270 Madrid, Spain; jgherrer@inf.uc3m.es (J.G.H.); molina@ia.uc3m.es (J.M.M.L.)

**Keywords:** LSTM, filtering, forecasting, regression, encoder–decoder, attention, system identification, deep learning

## Abstract

Certain difficulties in path forecasting and filtering problems are based in the initial hypothesis of estimation and filtering techniques. Common hypotheses include that the system can be modeled as linear, Markovian, Gaussian, or all at one time. Although, in many cases, there are strategies to tackle problems with approaches that show very good results, the associated engineering process can become highly complex, requiring a great deal of time or even becoming unapproachable. To have tools to tackle complex problems without starting from a previous hypothesis but to continue to solve classic challenges and sharpen the implementation of estimation and filtering systems is of high scientific interest. This paper addresses the forecast–filter problem from deep learning paradigms with a neural network architecture inspired by natural language processing techniques and data structure. Unlike Kalman, this proposal performs the process of prediction and filtering in the same phase, while Kalman requires two phases. We propose three different study cases of incremental conceptual difficulty. The experimentation is divided into five parts: the standardization effect in raw data, proposal validation, filtering, loss of measurements (forecasting), and, finally, robustness. The results are compared with a Kalman filter, showing that the proposal is comparable in terms of the error within the linear case, with improved performance when facing non-linear systems.

## 1. Introduction 

Many problems in engineering and research require or are based in forecasting or filtering parameters along time, understood by forecasting the predicted values for future times in the sequence. These processes are often associated with sensor-recorded values with a certain degree of accuracy. When the noise level has been reduced from the desired parameters, this is a filtering case.

The problems of estimation and filtering are not new, a classic study field is the theory of stochastic observers. The Aström [1] and Lewis [2] books provide an introduction into stochastic estimator theory and have been referenced in thousands of publications. Classical estimation methods have innumerable successful applications and continue to be one of the starting points for estimation and filtering problems. For an overview of classical and Bayesian estimation techniques, H. H. Afshari et al.’s [3] work provides a systematic review of all classical and Bayesian estimation techniques and their possible applications.

One of the principal landmarks in stochastic observer theory is the optimal stochastic estimators formulation or Kalman filter (KF) [4,5,6]. These estimators are based in the state space systems and different versions, such as extended KF (EKF) [7,8,9], unscented KF (UKF) [10,11], or robust KF (RKF) [12], generalize its use with nonlinear Gaussian problems as shown in Afshari et al. [3]. However, sometimes the systems can present complexities that may be unapproachable from a classical perspective. In other cases, the systems present behaviors with memory (non-Markovian), like people moving around among other people [13]. In these cases, classical solutions provide approximations that diverge from the wanted behavior.

The KF is a widely used system for filtering and state estimation. This estimator uses linear systems and Gaussian noise as starting assumptions to find a feedback gain (Kalman gain) that exponentially minimizes the system covariance. On the other hand, the systems that can be solved by Kalman or its extended version, EKF, are Markovian, in other words, for state estimation they only use contiguous states but without taking into account the behavior (states) at other times. This limits the use in problems with context, such as natural language processing or human behavior, among others.

In the face of these limitations, artificial intelligence paradigms provide an interesting opportunity to study. It is interesting how hybrids between classical and artificial intelligence systems have been achieved, such as those made by Satish. R et al. [10] or H. Caskun [14]. In [14], a neuronal estimator was fused with a KF for human image pose regularization. Works such as J. Mohd et al. [15] used the term “software sensors” to describe computational algorithms to estimate system states that are complex to measure, expensive, or non-observable. Thus, computational artificial intelligent (AI) techniques were shown to be an alternative to classical estimators in the face of certain problems. In this line we can find many works, such as those of [15,16], in which they use several features of the input in their models.

New perspectives in machine learning techniques address several classical theories limitations problems as shown in Park’s work [17]. Park modeled the potential trajectories of nearby vehicles from a grid that formed an occupation map and an encoder–decoder system based on long short-term memory (LSTM) cells. If we know the states to be estimated or modeled, we can find problems with time series estimation or systems modeling.

Time series forecasting works, to some extent, to identify/model the dynamical system that the observations describe. The LSTM cells architectures have proved their potential in front of traditional techniques, such as ARMA (autoregressive moving average), SARIMA (seasonal autoregressive integrated moving average model), and ARMAX (autoregressive-moving average with exogenous terms). A good example of this is Muzaffar and Afshari’s work [18], where they compared the previous traditional techniques with a light LSTM architecture for the electric charge estimation case in ranges of different time sampling, under root mean squared error (RMSE) and mean absolute percentage error (MAPE) metrics, where the LSTM architecture showed better results than the traditional techniques in several experiments, and this proposed system is very susceptible to improvements to increase the performance.

Deep learning (DL) in forecasting, filtering, or classification problems attempts to fit internal network functions to an input data set to make inferences. Relying on the architecture of the neural network, the cost function, the training algorithm, hyperparameters, and especially the dataset, the network can be adapted to a greater or lesser extent to the desired output.

While Kalman seeks to minimize its covariance based on prior assumptions, a deep neural network does not assume any of Kalman’s assumptions but attempts to adapt its hidden dynamics to the training data independently of their distribution or the dynamical relationship between them. This neural network flexibility provides an opportunity to generalize estimation and filtering problems under artificial intelligence paradigms.

A previous work [19] made a first approach to forecasting and filtering problems in an increasing linear dynamic system with noisy measurements from a DL perspective. In [19], the authors highlighted the neural network saturation problem in non-bounded system estimation. To solve this problem, a recursive data standardization method based on overlapping sliding windows and a neural architecture with LSTM cells is proposed.

This paper tackles the forecast-filtering problem of trajectories from deep learning paradigms. We propose a novel method of network density adjustment based on J. Llerena et al.’s work [19]. That method generalized the estimation and filtering problem without any initial hypothesis about the system or measurement type (linear or nonlinear, Markovian or non-Markovian, or Gaussian or non-Gaussian), performing a rigorous analysis of the problem and solutions with a high experimental burden to evaluate the estimator performance.

Unlike Kalman, this proposal performs the process of prediction and filtering in the same phase, while Kalman requires two phases. In this evaluation, we study three different dynamic system trajectories. We selected a set of systems with a progressive transition for the reader, starting from the position estimation in a uniform rectilinear motion (URM) in 1D (Section 4.1); next, a sinusoidal paths of a 1D object (Section 4.2); and finally the curved trajectories defined by a nonlinear dynamic model described by the Volterra–Lotka evolutionary equations (Section 4.3). The proposed neural estimator is evaluated for different cases under five experiments: data preprocessing effect on database (Section 4.4.1), filtering with complete sequences (Section 4.4.2), recursive filtering with new measurements (Section 4.4.3), loss in measurement estimation simulation (Section 4.4.4), and finally the impact on the filtering when receiving measurements far from the model (Section 4.4.5). 

The neural estimators proposed are supported by an encoder–decoder system based on natural language processing methods, which increases its depth with the complexity of the systems.

Finally, the contributions of the present work can be summarized in the following items:

- An approach has been developed to adapt a neural architecture previously used for natural language processing to the specific problem of estimation and filtering without needing the previous hypotheses about the type of system.
- The proposed method shows a comparable performance in terms of error with respect to KF in linear systems, while in the case of nonlinear systems it shows its potential to improve in terms of error and robustness.
- The principal advantages of our method lies in the simplicity of the neuro-estimator/filter as a model building learnt from data with respect to KF.
- The proposed method can address estimation and filtering problems for linear, nonlinear, Markovian, non-Markovian, Gaussian, and non-Gaussian systems.

This paper has been organized as follows: in Section 2, we define the problem and introduce how to approach the problem from a classic observer’s perspective and a review of possible solutions to estimation and filtering problems from deep learning paradigms. In Section 3, we describe the proposal of study, the methodology for its realization, and a rigorous mathematical definition. Section 4 includes details of the three case studies based on the proposal and the proposed set of experiments. Finally, in Section 5, our conclusions are presented.

## 2. General Problem Formulation

We consider an unknown dynamic system f not necessarily linear or Markovian. From this system we only know noise measurements z of trajectories described from observable system states x in time t. Measurements z are connected with the system states by the h function. Generally, h can be considered nonlinear and dependent of a stochastic parameter v(t).

(1) $$\frac{dx\left(t\right)}{dt}=f\left(x\left(t\right)\right)$$

(2) z(t)=h(x,v)=h(x)+v

Here, $x\left(t\right)\in {\mathbb{R}}^{n}$ is the state vector, f is a state vector field, and h is a function that selects a subset of specific states. If f is of the Lipchitz type, it is possible to transform the continuous-time problem to a discrete-time one:

(3) $$x_{k+1}=F^{*}\left(x_{k}\right)=x_{k}+\int_{t_{k}}^{t_{k+1}}f\left(x\left(\tau \right)\right)d\tau$$

A common way to discretize generally linear systems is to use the approximation $\dot{x}=\frac{x_{k+1}-x_{k}}{T_{s}}$, where $T_{s}$ refers to the sampling time that we can also find as ΔT or T.

Removing the assumption of a Markovian system, the future states not only depend on the previous instant states $x_{k}$, but also have long-term temporal dependencies, and thus we can formulate it as follows:

(4) $$x_{k+1}=F^{*}\left(x_{k}\right)=\int_{t_{l}}^{t_{k+1}}f\left(x\left(\tau \right)\right)d\tau$$

where $t_{l}$ is a temporal instant less than k and generally unknown in non-Markovian systems, where the approach for the previous discretization can no longer be used. In this way classical dynamic system can be considered as a particular case of a non-Markovian system.

According to this notation, the forecasting state problem is formulated in relation to the previous states (4), which means that the forecasting consists of identifying states in future times ($x_{k+1}$). On the other hand, a filtering problem base identifies certain $x_{k}\;$states at the same moment in which $z_{k}$ noise measurements are received (5).

(5) $$x_{k}=h^{-1}\left(z_{k}-v_{k}\right)$$

However, in real problems, it is not possible to know the noise value, $v_{k},$ and the h function may not be invertible, so that the state vector has to be estimated from observations. If we name ${\hat{F}}^{+}$ and $\hat{F}$ the filtered and predicted estimators, respectively, the problem is how to generate these estimators from observations:

(6) $${\hat{x}}_{k}=\;{\hat{F}}^{+}(z_{0},\;\ldots ,z_{k-1},{\;z}_{k})$$

(7) $${\hat{x}}_{k+1}=\hat{F}(z_{0},\ldots ,\;z_{k-1},\;z_{k})$$

The objective of this process is to build the estimators with the minimum error from the ideal values. 

### 2.1. Kalman Solution

In Bayesian estimation theory, KF is the optimal solution for a linear dynamic system and Gaussian noise in the measurement and estimation process [1,2]. For a stochastic nonlinear dynamic system (8), the first approximation derived from the KF is the EKF.

(8) $$\dot{x}=f\left(x,u,w\right)\;z=h\left(x,v\right)$$

As in the linear KF [1,2,3], w shows the noise process and v shows the measurement noise. The system and measurement model can be nonlinear. The EKF idea is built around the linearization system over the estimated states $\hat{x}$. This means that f and h must be derived with respect to the states x, the model noise w, measurement noises v, and the input signal u. In our case, we considered an autonomous system:

(9) $$A={\nabla f\left(x,0,0\right)|}_{\left(\hat{x},u,0\right)}\;W=\nabla f{\left(0,0,w\right)|}_{\left(\hat{x},u,0\right)}\;H={\nabla h\left(x_{k},0\right)|}_{\left(\hat{x},u,0\right)}\;V={\nabla h\left(0,v\right)|}_{\left(\hat{x},u,0\right)}$$

The first bracket in the previous equations refers to the terms with respect to the functions derived from the system and measurements, while the second bracket refers to the values to be substituted in our Jacobian matrix.

The matrices A, W, H, and V are the equivalent to the linearized f, h system. A is the linear system matrix, H is the observation matrix, W is the process noise, and V is the observation noise, all in continuous space. If the system has an input signal u, we can find the input matrix B and the direct transmission matrix D; however, in autonomous systems, these matrices do not exist. When discretizing a linear continuous system to discrete space with a sampling time ∆T, some of the above matrices traditionally acquire another notation symbol: A→ ϕ and B→Γ.

When the continuous system has been linearized, the next step is to discretize and apply the same process as in the linear KF. This classical theory decouples, in two different phases, the problem of prediction and filtering.

Kalman filters and EKF have two steps, prediction and update. To identify these steps and the temporary state, Kalman notation uses a sub-index in the form $x_{\gamma |\delta }$. The first, γ, refers to the temporal state (current = k and previous = k−1) and the second, δ, refers to the filter step (prediction = k−1 and update = k).

The KF step formulation is formulated as follows when the system does not have noise in the estimation process and is autonomous when Γ=0 or when the control signal $u_{k}=0$.

***Prediction step:***

(10) $${\hat{x}}_{k|k-1}=\phi {\hat{x}}_{k-1|k-1}\;P_{k|k-1}=\phi P_{k-1|k-1}\phi^{T}+Q_{k}$$

***Update step:***

(11) $$G_{k}=P_{k|k-1}H^{T}{\left(HP_{k|k-1}H^{T}+R_{k}\right)}^{-1}\;{\hat{x}}_{k|k}={\hat{x}}_{k|k-1}+G_{k}\left(z_{k}-H{\hat{x}}_{k|k-1}\right)\;P_{k|k}=\left(I-G_{k}H\right)P_{k|k-1}$$

In this way, both problems with forecasting and filtering in Kalman are decoupled. In the Kalman case, the forecast is made on the current state k; thus, it is usually called prediction in place of forecast. First, a state space model (SSM) predicts the current time state vector ${\hat{x}}_{k|k-1}$ (prediction step), and then the prediction is improved ${\hat{x}}_{k|k}$ (current state vector in update step) with the current measure vector $z_{k}$.

The KF aim is to find a feedback gain G (optimal Kalman gain) that allows us to exponentially minimize the covariance P matrix (measure of the estimate accuracy) taking into account the covariance of the process noise Q ($W_{k}\sim N\left(0,Q_{k}\right)$) and the covariance of the measured observations R ($V_{k}\sim N\left(0,R_{k}\right)$), under the assumption that all noises are Gaussian, uncorrelated, and zero-mean.

### 2.2. Deep Learning Solutions

Many works related to forecasting or filtering problems can be found in the literature under system modeling, filtering/reconstruction, and prediction keywords around deep learning paradigms. In system identification we can highlight works related to the resolution of ordinary differential equations, such as that of Chen et al. [20]. Solving these equations lets us move through the state space that defines a dynamic system at the instant of time desired—in other words, predict the future states of the system or reconstruct them.

Some of the works on system modeling, such as Sierra and Santos [21], compare traditional techniques versus neural networks highlighting the relevance of using neural networks when the mathematical modeling is complex. Modeling solutions have been found that are robust to noise in the data. Rudy’s [22] work proposes a new modeling paradigm that simultaneously learns the dynamics of the system and the noise estimate of the measurements in each observation, managing to separate additive noise in the observations of the states of different systems.

Artificial neural networks (ANNs) for the modeling of nonlinear dynamical systems have proven to be a relevant solution. In Raissi [23], the performance of a neural system for the modeling of different nonlinear dynamic systems starting from synthetic data. The data refer to a time series describing the states of the systems under study. In this study, they used a simple neural architecture and compared the error of the predicted trajectories versus the density and depth of the neural networks, concluding that a deeper and denser network will not always show better results.

In the case of signal filtering, the Arsene work [24] showed a performance comparison in electrocardiogram (ECG) signal filtering between two deep learning filters with the two most popular trends at present, convolutional neural networks (CNNs) and LSTM, versus wavelet filters. Finally, the CNN architecture achieved better performance than the LSTM and the wavelet filter, but the proposed LSTM architecture can be improved.

When the systems to be predicted show non-Markovian behavior, SSM are not suitable. A widely studied set are those related to natural language processing.

Different studies regarding natural language processing with deep learning provides exportable tools to other study areas. In relation to this work, we can remark on the encoder–decoder architectures or the attention models. Y. Zhu et al. [25] showed a novel comparative study of different LSTM encoder–decoder architectures and attention mechanisms. Finally, they proposed a combined method of an encoder–decoder with attention mechanisms and LSTM cells for prediction. They used two different datasets, from the Alibaba Open Cluster Trace Program and Dinda workload dataset. Finally, the experiments showed that their proposed model achieved state-of-the-art performance.

The common link between several of the above studies lies in the intention to extract time trends from data sets with LSTM neural cells. LSTM neural cells are not new [26], but they have proven to be powerful in catching long short temporal dependencies in multiple examples. This is the reason for its use in other than recurrent architectures, such as gated recurrent unit (GRU), bidirectional-LSTM (BI-LSTM), or bidirectional encoder representations from transformer (BERT) architectures, used with great success as a new context extraction technique in natural language processing, as shown in J. Delvin’s paper [27].

The LSTM is an recurrent neural network (RNN) that allows long-term dependencies and overcomes the vanishing gradient issue [28]. Considering the relevance of this layer, detailed information of its structure can be found in works, such as those of [16,25,26], and [29,30,31,32]. In X. Song [16], we can see a typical structure of a LSTM layer versus a traditional recurrent network layer. Each cell of the LSTM layer is composed of different functions as shown in Y. Liu [32]. The processes that an LSTM cell performs when it receives new data are described as follows.

Given an input $x_{k}$ at time instant k and the hidden cell state h, the basic operation involves different sections of the neural cell, forget gate (12), input gate (13), candidate (14), and output gate (15). The hidden state h gives the LSTM cell the property to acquire memory, and this memory provides the opportunity to address non-Markovian problems. The forget gate $f_{k}$ decides which information $c_{k-1}$ is removed from the previous cell state. The input gate is responsible for identifying the input information $x_{k}$, which should be kept in the candidate memory cell ${\tilde{c}}_{k}$. The current memory vector $c_{k}$ is updated by linking the past information $c_{k-1}\;$with the candidate information ${\tilde{c}}_{k}$ (14). Finally, in the output gate (15), the hidden state $h_{k}$ cell is confirmed with the cell state $c_{k}$ and the $o_{k}$ output information.

(12) $$f_{k}=\sigma \left(x_{k}U_{f}+h_{k-1}w_{f}+b_{f}\right)$$

(13) $$i_{k}=\sigma \left(x_{k}U_{i}+h_{k-1}w_{i}+b_{i}\right)\;{\tilde{c}}_{k}=\tanh\left(x_{k}U_{c}+h_{k-1}w_{c}+b_{c}\right)$$

(14) $$c_{k}=f_{k}c_{k-1}+i_{k}{\tilde{c}}_{k}$$

(15) $$o_{t}=\sigma \left(x_{t}U_{o}+h_{k-1}w_{o}+b_{o}\right)\;h_{t}=o_{t}*tanh\left(c_{t}\right)$$

Here, U is the input weight, W is the recurrent weights, and b is the bias. Subscripts represent the gates: f=forget,i=input,  c=candidate, and o=output. The activation function σ is the sigmoid function, and tanh is the hyperbolic tangent function. The first function is bounded between 0 and 1, and tanh between −1 and 1.

All the above cases are grouped under a regression problem in which the objective is to optimize/adjust the network function ${\hat{F}}_{\theta }$ to the Φ dataset. To fit ${\hat{F}}_{\theta }$ to the Φ dataset, the ${\hat{F}}_{\theta }$ function must be parameterized (θ) with a cost function ℒ(θ) to be optimized and an optimization methodology, where ${\hat{F}}_{\theta }$ means a network function parameterized by the internal θ terms. These internal parameters are the weights and biases of each internal neuron.

As S. Rudy et al. showed in [22], we can mathematically define a recurrent neural network as the composition of $g_{i}$ functions that define each i-layer of the network. In addition, these $g_{i}$ functions are the result of the composition of the $s_{j}$ functions that define each neuron.

(16) $${\hat{F}}_{\theta }\left(x\right)=\left(\prod_{i=1}^{l}C_{g_{i}}\right)\left(x\right)$$

Here, $g_{i}\left(x\right)=\left(\prod_{j=1}^{N_{i}}C_{s_{j}}\right)\left(x\right)\;|\;s_{j}=\;\sigma_{j}\left(xW_{j}+b_{j\;}\right)$ is an i-layer function. $C_{s_{j}}$ is the s composition operator for each j activation function $\sigma_{j}:\mathbb{R}\to \mathbb{R}$ and $\theta ={\left\{W_{i\;},b_{i\;}\right\}}_{i=1}^{l}|W_{i\;}\in \;{\mathbb{R}}^{N_{i}\times N_{i-1}},\;b_{i\;}\in \;{\mathbb{R}}^{N_{i}}$ is the network parameterization function in terms of its weights $W_{i\;}$and biases $b_{i\;}$. $N_{0},N_{1},\ldots ,N_{l}$ are the number of neurons in each layer, where $N_{0}=d\;|\;d\in \mathbb{N}$ is the input layer and l∈ℕ is number of network layers. The term ^ over the F function means “estimated”, which is inherited from the classical notation from stochastic observers.

Taking LSTM cells in different layers, we must take into consideration the weights associated with the internal states $U_{\partial ,i}$ and transitions of the LSTM cells. Finally, the parameter network functions are: $\theta ={\left\{U_{\partial ,i},W_{\partial ,i\;},b_{\partial ,i\;}\right\}}_{i=1}^{l}$ where $W_{\partial ,i\;}\in \;{\mathbb{R}}^{\partial \times N_{i}\times N_{i-1}},\;b_{\partial ,i\;}\in \;{\mathbb{R}}^{\partial \times N_{i}}$ and spreading typical LSTM notation  ∂≡{forget=f, input=i, output=o, candidate=c, and non_LSTM_gate=n}.

## 3. Proposal Formulation 

In this paper, we proposed to approach the joint problem as forecasting-filtering trajectories without assuming a hypothesis of linear, Markovian, or Gaussian behaviors, based only on supervised information and in only one processing stage to build the estimator ${\hat{x}}_{k+1}$ from the available observations, $\;z_{k},z_{k-1},\;\ldots z_{k-L}$ based on a model built with representative training data.

(17) $$x_{k+1}={\hat{F}}^{*}\left(x_{k}\right)={\hat{F}}^{*}\left(h^{-1}\left(z_{k}-v_{k}\right)\right)=\hat{F}\left(z_{k}\right)$$

For this purpose, the recursive method with overlapping sliding time windows with Llerena et al.’s work [19] Algorithm 1 is combined with the artificial neural architecture configuration process of Algorithm 2. The general process can be seen at a high level in Algorithm 1. The overlapping region between windows is used to activate the network, with activation being understood as a period for initializing the network to update its hidden states. This allows the network to activate its internal long-term memory with which to recall time trends of data from the previous time window. We had two cases of initialization, during the first-time window (no overlap window yet), lines 6–8 in Algorithm 1 and, when overlaps between adjacent windows happen, lines 9–10. In the first case, as new measurements are received, they are piled up in an S-sequence until the size of the overlay/activation is defined as O. In the second case, the last measurements received in the previous time window are recycled to activate the network during the second (and successive) time windows.

The method makes it possible to address problems with continuous measurements in a recursive manner and also when a measurement is lost. If we look at the general process of Algorithm 1 line 12–20, in the case of not receiving new measurements, the system uses the previous filtered estimation to feed the network and obtain the following state.

**Algorithm 1.** General proposal process.

**1:**

L = sliding time window length

**2:**
O = overlap window length (activation area) 
**3:**
**procedure** GENERAL PROCESS $\left(L,O,z_{k}\right)$

**4:**

**for**
k=1→L

**5:**
 ***If*** start & 1st sliding window
**6:**
  **While** Nº measurements < O
**7:**

$\;\;\;S_{k}=z_{k}$

**8:**
   **end while**
**9:**
 **else if** start
**10:**
  S = $\left[z_{L-O},z_{L-O+1},\;\ldots z_{L}\right]$

**11:**
 **else**
**12:**
  **If** new measure
**13:**

$\;\;\;S=z_{k}$

**14:**
   S→ standardization$\to S^{*}\to$Net & update internal states$\to {\hat{x}}_{k+1}^{*}$

**15:**

$\;\;\;{\hat{x}}_{k+1}^{*}\to unstandardization\to return\left({\hat{x}}_{k+1}\right)$

**16:**
  **else**
**17:**

$\;\;\;S^{*}=x_{k}^{*}$

**18:**
$\;\;\;S^{*}\to$Net & update internal states$\to {\hat{x}}_{k+1}^{*}$

**19:**

$\;\;\;{\hat{x}}_{k+1}^{*}\to unstandardization\to return\left({\hat{x}}_{k+1}\right)$

**20:**
  **end If**
**21:**
 **end if**
**22:**

**end for**

**23:**
Move sliding window L-O & Start again
**24:**

**end procedure**


For this, three main blocks are differentiated: the generation of a synthetic database that allows us to control the system’s performance, network building, and training, and finally inference with the trained network, like Algorithm 1 shows. 

The key to the generation of the synthetic database Φ, lies in matching noisy trajectories with ideal trajectories shifted one-time unit under $\Phi_{i}$ data packages. The noise paths $Z_{i}$ are generated by adding a Gaussian noise with $R_{k}$ variance to the simulated system states paths $X_{i}^{*}$ to be measured. If the measured paths $Z_{i}$ start at $z_{0}$ and end at $z_{k}$, the target paths $X_{i}^{*}$ start at $x_{1}^{*}$ and end at $x_{k+1}^{*}$, thus, maintaining the dimensionality one unit shifted. The size of the time window is therefore the L values. The length of the simulated trajectories is equal to two consecutive non-overlapping time windows, so that the first-time window of each trajectory is used for the training subset and the second for the validation subset. Thus, the problem is formulated as a sequence-to-sequence learning system.

To make step-by-step inference, a neural architecture is composed of LSTM cells. These neuronal units take advantage of their internal states as a memory to be able to relate measurements to previous and later states, allowing inferences from sequence to sequence, sequence to step, and step to step.

We assume, for this purpose, the neural network function ${\hat{F}}_{\theta }$ can be adapted to a function F that defines a dataset Φ, where θ are the internal network parameters. The ∧ symbol over $F_{\theta }$ is inherited from the classical estimator’s notation. 

Then, the problem is to identify the parameters θ of an ANN using exclusively supervised information, as in [19], which associates $\Phi_{i}$ packages of $Z_{i}$ noise system paths with ideal $X_{i}^{*}$ paths states.

(18) $${\hat{F}}_{\theta }\left(z_{k}\right)\approx F\left(z_{k}\right)$$

### 3.1. Artificial Neural Network Architecture

The general network architecture proposed in Llerena et al. [19] consists of an encoder–decoder system based on good results with non-Markovian system models like [18,23,32]. Other fundamentals of design of this architecture focus on filtering problems, such as [24] or the identification of noisy systems [22,23]. The encoder and decoder are composed of LSTM recursive structures. Using LSTM layers, it is possible to extract long-term and non-Markovian trends and show their potential in estimation problems [16,33,34,35]. However, other types of dynamic systems have other particular conditions of information or number of measured states, and the architecture proposed in [19] does not have to be suitable with all systems; thereby, Algorithm 2 proposes a configuration method to adapt [19]’s neural architecture to a specific case.

Starting from the structure proposed in [19], focused on the benefits in front of regression problems of each one the layers and proven performance in URM paths, we propose an algorithm in Algorithm 2 to increase the depth of the encoder and decoder to adapt the results in front of other paths that are likely more complex in learning terms compared with URM paths.

Finally, at the output network side, we added a regression layer to implement the cost function ℒ(θ) (19) used to train the network system. Depending on the variability of the training set and the complexity of the system, the depth of the encoder–decoder and, in general, the network density must be adapted to obtain good training results.

**Algorithm 2.** The network architecture configuration process.

**1:**
Sliding time window dimension selection
**2:**
J. Llerena [19] Architecture adaption
**3:**
Width = number of features
**4:**
**procedure** ADAPT NETWORK TO SPECIFIC SYSTEM
**5:**

**train**
loos, MSE measure

**6:**

**while**
RMSE (net)>>RMSE(data)

**7:**
  **switch**
Nº iteration

**8:**
  **case 1:**
**9:**
  Hidden encoder and decoder layer = number of data whit sliding time window
**10:**
  **case 2:**
**11:**
  Increase number of units in the interconnexion layer.
**12:**
  **otherwise**
**13:**
  Add new LSTM layer in encoder with half hidden units than previous LSTM layer
**13:**
  **end switch**
**14:**
  **goto**→train
**15:**

**else**

**16:**
Save trained network
**17:**

**end while**

**18:**

**end procedure**


### 3.2. Computational Neural Network Framework

Under the supervised learning paradigms, we found that our problem consisted of identification systems or the regression problem. We can consider this problem as an optimization problem where we attempt to minimize the cost function ℒ(θ) by modifying the internal θ parameters from function ${\hat{F}}_{\theta }$ that we want to identify/adjust from the Φ dataset. The typical cost function ℒ(θ) is the means square error (MSE). When we take the derivative of the MSE used in the updating the parameters during the backpropagation, the value 2 of the power can be cancelled if the term $\frac{1}{2}$ is added to the MSE. Thus, the mathematical arrangement for the definite cost function is obtained and called the half means square error (HMSE). To control for possible overfitting effects, an $L_{2}$ regularization is added to the net weights, with λ being the regularization factor.

(19) $$ℒ\left(\theta \right)=\frac{1}{2S}\overset{S}{\underset{k=1}{\sum }}\overset{R}{\underset{j=1}{\sum }}{\left(X_{kj}^{*}-{\hat{F}}_{\theta }\left(Z_{k,j}\right)\right)}^{2}+\lambda \overset{l}{\underset{i=1}{\sum }}\overset{N_{i}}{\underset{j=1}{\sum }}\|\theta_{j}\|{}_{2}^{2}$$

S is the sequence length and R is the number of sequence parameters. On the other hand, this can be found in the literature [36,37,38,39], as the addition of Gaussian noise in the input data helps the regularization the network, for example with Tikhonov regularization [40]. Thus, using z-data with a certain level of $N\left(0,\sigma^{2}\right)$ noise also helps the regularization effect in the network.

As an optimization methodology, the Adam algorithm is used, which has amply demonstrated its performance with recurrent neural architectures as can be seen in the comparison with other algorithms in Kingma and Lei’s work [41].

Unlike Kalman, our system does not require Gaussian noise distribution, as the cost function does not assume any distribution. In addition, the network or cost function does not need to assume the system is linear, because the network function is fitted to the data behavior.

## 4. Case Studies and Experimentation

The following shows different case studies. For each one, we describe the synthetic data generation model, the classic estimator model and the neuronal structure used. All of them are accompanied by the configurations to help reproduce the results.

Among the classical estimators, KF is the optimal solution in the case of linear dynamical systems with Gaussian noise. When the system is not linear, its first approximation, EKF, is a widely extended method. To facilitate the comparison of our solution with the KF as a reference system in the experimentation, the measurements are simulated with Gaussian noise.

For each study case, we conducted the following experiments:

1. Standardization effect.
2. LSTM model validation and filtering comparison.
3. Filtering system simulation with new measurements along the first and second-time window.
4. Simulation of missing measurements in the input to filtering system; we estimated in the first and second-time window on a signal test, applying only measurements in the overlap section, first window, and first window measurements for the second case.
5. Impact on filtering of measurements generated with parameters far from the design.

The first experiment was used to visually check that the data converted to the standardized space remained bounded. The systems were evaluated in filtering and estimation. The RMSE was used as an evaluation metric in different ways. For complete sequences, we used experiment Section 4.4.2 with Equation (21) on each of the N validation trajectories over the k-time position associate with two different checkpoints. R is the number of states to be analyzed. If the system had a R>1, the RMSE was determined for each of the j states independently and in aggregate as the RMSE of the geometrical distance error $D_{i,k}$ (20). This can be seen in case study Section 4.3. With partial sequences, continuous feed data, and loss data, we used experiments Section 4.4.3 and Section 4.4.4. For these cases, Equation (22) was used as the evaluation metric, where L is the temporal size of the trajectories, R is the number of states, and O means the number of overlap data.

Experiment Section 4.4.5 tested the behavior of the systems in the face of new data deviating from the original design. The mean (24), median (25), and mode were used to evaluate the behavior with the RMSE (23) obtained from each of the N new trajectories obtained in each variation of the independent terms of the simulation systems. The mode of the ordered set E, will be the value $E_{i}$ with the highest frequency in E, where $E={\left\{E_{1}=\min_{i}\left(RMSE_{3}^{i}\right),E_{2},\ldots ,E_{N-1},E_{N}=\max_{i}\;\left(RMSE_{3}^{i}\right)\right\}}_{i=1}^{N}$.

(20) $$e_{i,k,j}=X_{i,k,j}^{*}-{\hat{X}}^{*}\left(Z_{i,k,j}\right);D_{i,k}=\sqrt{\sum_{j=1}^{R}e_{i,k,j}^{2}}$$

(21) $$RMSE_{1}=\sqrt{\frac{1}{N}\sum_{i=1}^{N}D_{i,k}^{2}};\;k=Checkpoint\;\left\{1,2\right\}$$

(22) $$RMSE_{2}=\sqrt{\frac{1}{L}\sum_{k=O+\;1}^{L}D_{k}^{2}}$$

(23) $$RMSE_{3}^{i}=\sqrt{\frac{1}{L}\sum_{k=1}^{L}D_{i,k}^{2}}$$

(24) $$Mean=\frac{1}{N}\sum_{i=1}^{N}E_{i}$$

(25) $$Median=\{\begin{array}{cc} E_{\left(N+1\right)/2} & if\;N\;is\;odd\\ \frac{1}{2}\left(E_{N/2}+E_{1+N/2}\right) & if\;N\;is\;even \end{array}$$

Equation (20) is the estimation error and geometrical distance error, where ${\hat{X}}^{*}\left(Z_{i,k,j}\right)$ can be Kalman ${\hat{X}}^{*}{}_{i,k,j|\;k}$ or LSTM ${\hat{F}}_{\theta }\left(Z_{i,k,j}\right)$, remembering that the superscript ∗ refers the subvector state to be estimated. The subscript i denotes trajectory i in a set of N trajectories, if the error (20) is calculated over a single trajectory, the term is removed as in (22). Finally, the subscript k is the time step, and j is the system state.

For the second experiment, we show a histogram of the estimation error of each test trajectories over the check points. If the system predicts R>1 states (case study Section 4.3), initially, this is shown as the error of each state and then the Euclidean distance between the ideal checkpoint $X_{i,k,j}^{*}\;$ and estimate system ${\hat{X}}^{*}{}_{i,k,j}$. Experiments Section 4.4.3 and Section 4.4.4 show the trajectory evolution and step-by-step error for specific initial conditions during two consecutive time windows. The error was determined for each state independently as in (20). Finally, experiment Section 4.4.5 shows the KF and LSTM mean, median, and mode evolution in Section 4.2 and Section 4.3 case studies as the independent terms of the trajectory simulation systems are changed.

To simulate each system trajectory, we used the Ode45 algorithm [42], while, for the estimation of states for each case study with Kalman techniques from the classical models, the formulation used is indicated in each of the systems. For training each ANN model, we trained over 80 epochs with 20 batches and an initial learning rate of 0.005. After eight epochs, we applied a 0.5 learning drop factor. Finally, we applied a $\lambda =10^{-4}$ $L_{2}$ regularization factor. 

All the algorithms were implemented on MATLAB [43]. The experiments were performed on a commodity machine with Windows 10 Home 64 bit hosted in Intel ^®^ Core™ i7-8550U CPU @1.80 GHz 1.99 GHz with 12 GB RAM and 512 GB SSD from internal memory, graphic card Nvidia GeForce 940MX 64 bits.

### 4.1. Linear Paths (Uniform Rectilinear Motion) 

The model of linear paths is associated with a 1D uniform rectilinear motion, composed of the states of position p and speed v. To simulate state measurements, we only considered the position H=[1 0] under gaussian noise $V_{k}\sim N\left(0,\sigma_{p}\right)$. The simulated paths consider the ideal model, without process noise $W_{k}=[0{,0]}^{T}$.

(26) $${\left[\begin{array}{c} p\\ v \end{array}\right]}_{k}=\left[\begin{array}{cc} 1 & T\\ 0 & 1 \end{array}\right]{\left[\begin{array}{c} p\\ v \end{array}\right]}_{k-1}+W_{k}$$

(27) $$z_{k}=Hx_{k}+V_{k}$$

The synthetic data is generated with Table III as described in Llerena’s work [19].

#### 4.1.1. Classical State Estimator

As an estimator, we used a linear KF. In this case, the process noise is $W_{k}=[0{,0]}^{T},\;$and the position measurements have Gaussian noise $N\left(0,\sigma_{Z}\right)$ (27), as in Algorithm 1 described in Llerena’s work [19]. The system model corresponds with Equation (26) and Algorithm 1′s parameters of [19]. KF requires two steps to obtain the immeasurable state (speed) as $v_{2}=\left(p_{2}-p_{1}\right)/T$ and initialize the covariance matrix start, like this: $P_{2|k-1}=\sigma_{Z}\left(\begin{array}{cc} 1 & 100\\ 100 & 2 \end{array}\right)$.

(28) $${\hat{x}}_{k|k-1}=\phi {\hat{x}}_{k-1|k-1}$$

#### 4.1.2. Artificial Neural Structure

As in work [19], the architecture referenced in Algorithm 2 of that work is used. This architecture is composed of an input layer with 80 samples and one feature. The encoder has 400 hidden units and the decoder has 200, both composed with LSTM cells. The interconnection layer between the encoder and the decoder corresponds to a fully connected layer with a rectified linear unit (ReLU) function.

### 4.2. Sinusoidal Paths (Simple Harmonic Motion)

To generate sinusoidal paths, we considered a 1D system with simple harmonic motion that defines the transversal position with constant amplitude and frequency. The system states are given by the position $x_{1}$ and the speed $x_{2}$.

(29) $$\left[\begin{array}{c} {\dot{x}}_{1}\\ {\dot{x}}_{2} \end{array}\right]=\left[\begin{array}{cc} 0 & 1\\ -\omega^{2} & 0 \end{array}\right]\left[\begin{array}{c} x_{1}\\ x_{2} \end{array}\right]+W$$

(30) z=Hx+v

To simulate state measurements, we only considered the first estate $x_{1}$, H=[1 0] under gaussian noise $V_{k}\sim N\left(0,\sigma_{x_{1}}\right)$. The simulated paths consider the ideal model, without process noise $W_{k}=[0{,0]}^{T}$.

The synthetic data is generated with Table 1 conditions:

#### 4.2.1. Classical State Estimator

Starting from Equations (29) and (30), using discretization (3) and applying Taylor’s series developments, finally our linear system is discretized as follows:

(31) $$\underset{{\hat{x}}_{k|k-1}}{\underbrace{{\left[\begin{array}{c} {\hat{x}}_{1}\\ {\hat{x}}_{2} \end{array}\right]}_{k}}}=\underset{\phi }{\underset{⏟}{\left[\begin{array}{cc} cos\left(\omega T\right) & \frac{\sin\left(\omega T\right)}{\omega }\\ -\omega sin\left(\omega T\right) & cos\left(\omega T\right) \end{array}\right]}}\overset{{\hat{x}}_{k-1|k-1}}{\overbrace{{\left[\begin{array}{c} {\hat{x}}_{1}\\ {\hat{x}}_{2} \end{array}\right]}_{k-1}}}$$

(32) $$z_{k}=Hx_{k}+V_{k}.$$

By assuming that we only measured the first of the states, we used the linear trajectory system strategy to find the second state and be able to initialize a filter in the third measure. As the estimator minimizes the covariance in an exponential way, the cross covariances can be made large to converge quickly, and this helps the new poles of the feedback system have a high negative real part:

(33) $$P_{2|k-1}=\sigma_{Z}\left(\begin{array}{cc} 1 & 1000\\ 1000 & 2 \end{array}\right)$$

#### 4.2.2. Artificial Neural Structure

Taking the method described in the process of Algorithm 2, the architecture proposed for the sinusoidal paths is the one indicated in Table 2.

### 4.3. Smooth Curved Paths (Volterra–Lotka System)

The proposed model to generate smooth curved paths is the Volterra–Lotka predator–prey model. This model indicates the evolution of two species parameterized with the growth rates of the prey $r_{1}$, the success of the hunt of the predator that affects the prey $a_{1}$, the growth rate of the predator $r_{2},$ and the success of the hunt that affects predator $a_{2}$. The paths used are those defined by the union of the two states, also known as phase diagrams.

This is an autonomous system that does not require any input or external signal u and presents a great variety of smooth curved paths in the whole of its state space.

We added a process noise term to the system $W={\left[w_{1},w_{2}\right]}^{T}={\left[0,0\right]}^{T}.$

(34) $$\{\begin{array}{c} {\dot{x}}_{1}=f_{1}\left(x,w\right)=r_{1}x_{1}-a_{1}x_{1}x_{2}+w_{1}\\ {\dot{x}}_{2}=f_{2}\left(x,w\right)=a_{2}x_{1}x_{2}-r_{2}x_{2}+w_{2} \end{array}$$

(35) z=h(x,v)=Hx+V

This system has an equilibrium point in $EP=\left[\frac{r_{2}}{a_{2}},\frac{r_{1}}{a_{1}}\right]$. Around this point, the system paths present a periodic evolution associated to a limit cycle attractor.

This study focuses on the set of initial conditions around 20% of the equilibrium point where the variety of trajectories is more pronounced.

The synthetic data is generated with Table 3 conditions:

#### 4.3.1. Classical State Estimator

Using the approximation of Equation (3), $\dot{x}=\frac{x_{k+1}-x_{k}}{T}$ the system is discretized as follows:

(36) $$\{\begin{array}{c} x_{1,k+1}=x_{1,k}+\;\left(r_{1}x_{1,k}-a_{1}x_{1,k}x_{2,k}+w_{1,k}\right)T\\ x_{2,k+1}=x_{2,k}+\left(a_{2}x_{1,k}x_{2,k}-r_{2}x_{2,k}+w_{2,k}\right)T \end{array}$$

(37) $$z_{k}=Hx_{k}+V_{k}$$

Since the system is non-linear, an EKF is formulated as an extension of the KF. In this way, the EKF is formulated with the following parameters:

(38) $$A={\nabla f\left(x,0\right)|}_{\left(\hat{x},0\right)}={\left(\begin{array}{cc} 1+\left(r_{1}-a_{1}x_{2}\right)T_{s} & -a_{1}x_{1}T_{s}\\ a_{2}x_{2}T_{s}\; & 1+\left(a_{2}x_{1}-r_{2}\right)T_{s} \end{array}\right)|}_{\left(\hat{x},0\right)}\;W=\nabla f{\left(0,0,w\right)|}_{\left(\hat{x},0\right)}=0_{2x2}\;H={\nabla h\left(x_{k},0\right)|}_{\left(\hat{x},0\right)}=I_{2x2}\;V={\nabla h\left(0,v\right)|}_{\left(\hat{x},0\right)}=V_{2x1}$$

We considered the system to be fully observable in which we could simultaneously measure the two states that we considered as positions on a two-dimensional plane, known in other environments under the phase diagram name. The measurement noise corresponds to a Gaussian noise $N\left(\mu ,\sigma_{z}\right)$ with mean μ=0 and variance $\sigma_{z}$.

(39) $$P_{1|k-1}=\sigma_{Z}I_{2x2}$$

#### 4.3.2. Artificial Neural Structure

Starting from the initial structure of the URM, the proposed structure for the Volterra–Lotka system is indicated in Table 4.

Although apparently the structure is similar to the URM, the density of the network is higher because it contains one more feature in the input and output layers, and a larger number of measurements to define the input/output layers.

### 4.4. Experimentation

In the following section, we show, in a compact way, each of the proposed experiments for the different study cases.

#### 4.4.1. Standardization Effect 

In this section, we show the dataset information mapping before and after applying the standardization process. We used the standardization process described in [19] based on [16].

**Figure 1:**
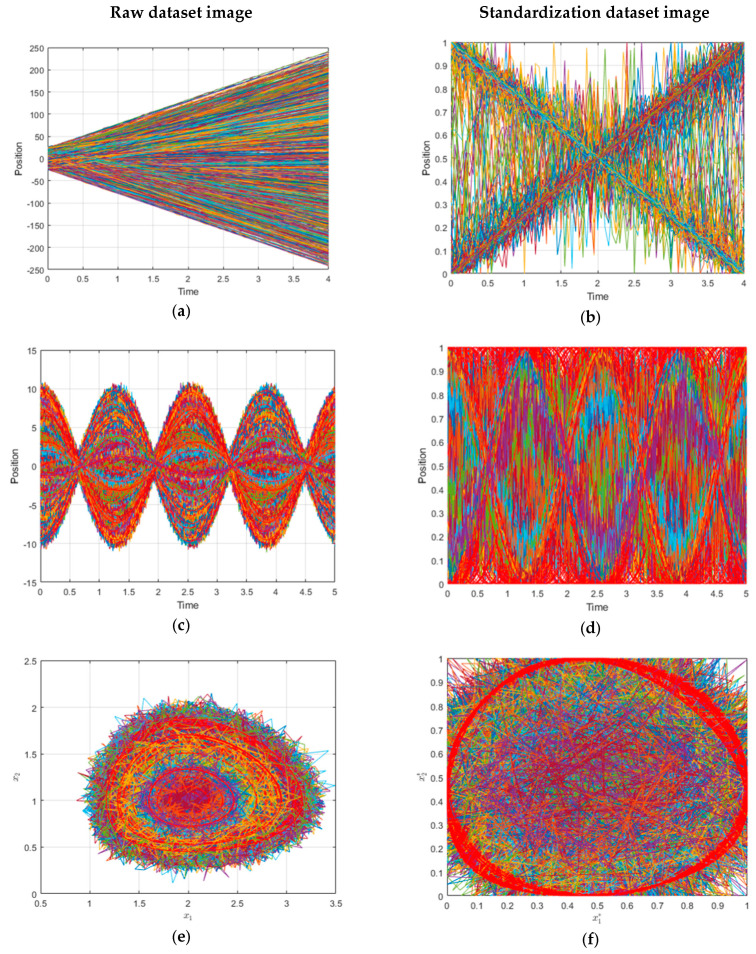
(**a**,**c**,**e**) a set of 10^3^ ideal paths in real space with uniform rectilinear motion (URM), sinusoidal, and Volterra System. (**b**,**d**,**f**) a set of 10^3^ paths in standardized space with URM, sinusoidal, and Volterra System.

First, it is important to emphasize that the arrival spaces after the standardization are bounded Figure 1b,d,f. Another perception that can be observed is that, for certain trajectories, the noise in the arrival space after the transformation can be attenuated (pronounced speeds, big amplitudes, or big closed paths) on the contrary increased (small speeds, amplitudes, and closed paths). This differentiation can be perceived by an intelligent system. These features combined with a bounded space are good hints to use ANN.

#### 4.4.2. Architecture Validation

The validation process of the different architectures is carried out using two checkpoints on each path. The first checkpoint is located just after the activation window and the second at the end of the data window. This is justified based on the KF covariance evolution, where it decreases exponentially in a linear system. Thus, KF will be less accurate at the beginning of receiving measurements than at the end.

**Figure 2:**
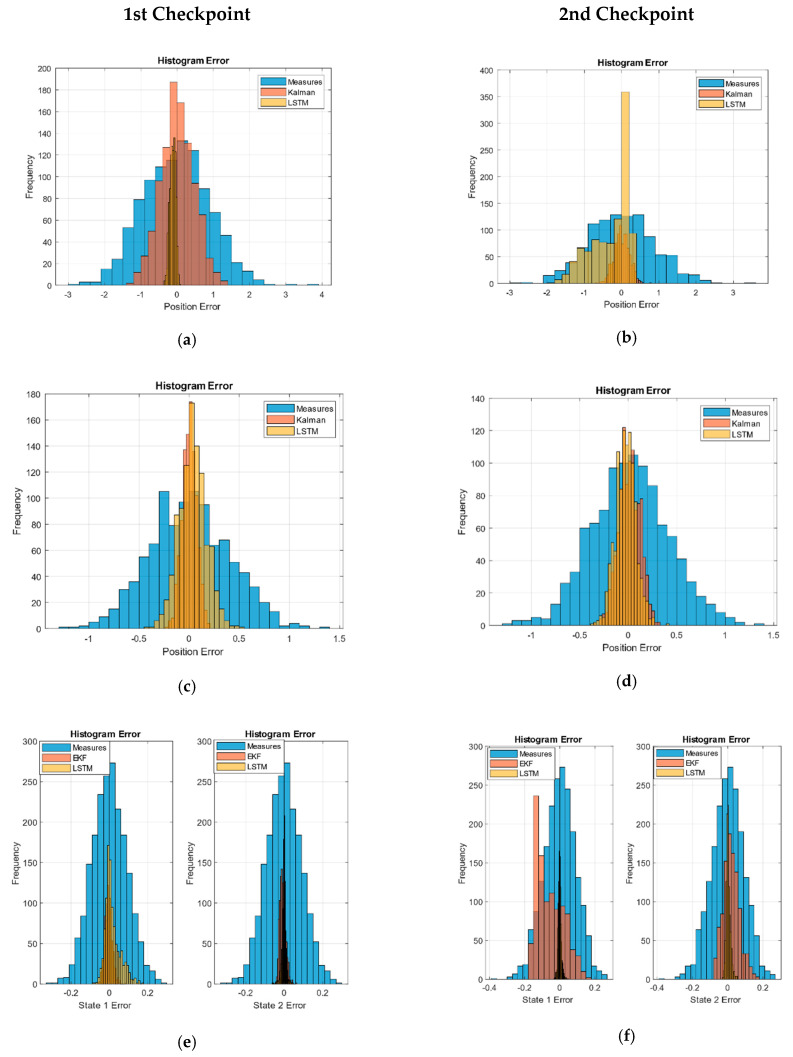

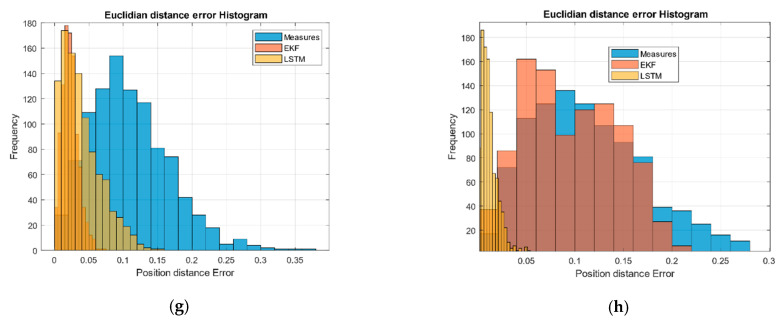
Long short-term memory (LSTM) and Kalman histogram validation: (**a**) first and (**b**) second, checkpoint in the URM model. (**c**) First and (**d**) second checkpoint in the sinusoidal path model. (**e**) First and (**f**) second checkpoint in the Volterra system paths. (**g**) First and (**h**) second checkpoint in the Volterra system (Euclidean distance error).

The checkpoints are taken over the measured, Kalman, and LSTM network outputs. The values obtained with each of the previous paths are compared with the ideal values, and the error value is saved. These errors are shown as a histogram in Figure 2, and the values of the RMSE obtained are shown in Table 5.

The error distributions of the sensor-measured data simulation show an invariant Gaussian behavior of the path position at the checkpoint. Given the nature of the RMSE, the values obtained correspond to the variance of the Gaussian noise.

We verified that the KF behavior implemented also presented a Gaussian distribution with less variance in the second checkpoint in linear systems cases (URM and sinusoidal). However, in the EKF case, we can see how the filter presents difficulties at the end of the paths but maintained the noise below the measurements.

In the case of the LSTM networks, we can see how the behavior was generally Gaussian except for the second checkpoint in the linear paths of the URM model. In the case of the second state of Volterra, it remained practically bounded, while in the sinusoidal trajectories, the first state of Volterra was reduced and was lower than in Kalman.

Figure 2g,h shows the system error as a Euclidean distance of the estimated XY positions with respect to the ideal values in order to check the deviation of the filter. All distributions have a tail to the right; however, this metric allows us to highlight the amount of data centered around the zero error. We verified how the performance of the LSTM network for this non-linear system showed great performance as the EKF approached. 

Finally, we verified how the proposed system with LSTM networks reduced the noise of the measurements and presented an error comparable to the KF.

#### 4.4.3. Filtering System Simulation with New Measurements

This experiment shows the behavior of Kalman and the proposed network when they are in continuous measurement feeds during the first and second time window when faced with a new set of data different from those used in the training and validation.

The initial conditions used in each system simulation are shown in Table 6. We used the same initial conditions for both experiments with continuous feed measurements and in the measurement experiment 4.4.4.

**Figure 3:**
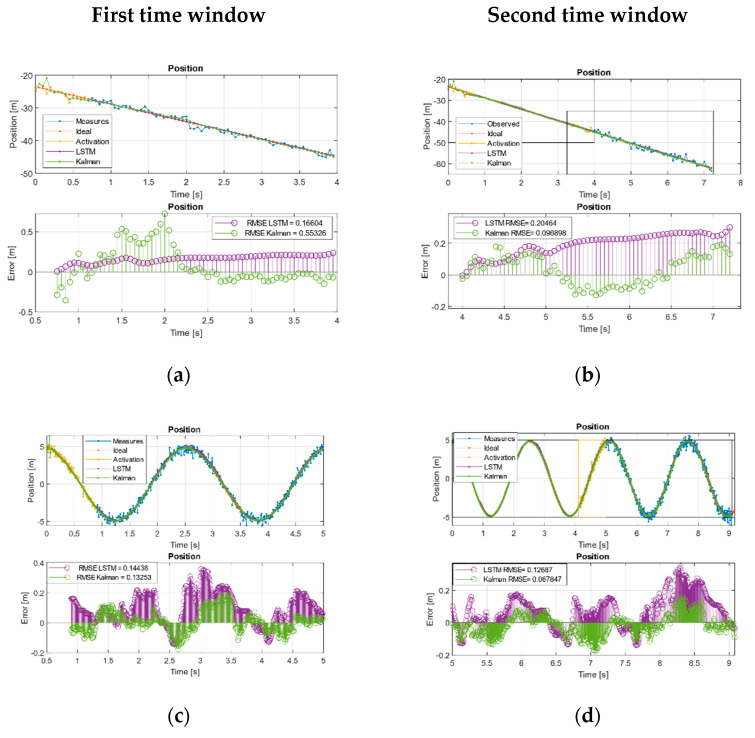

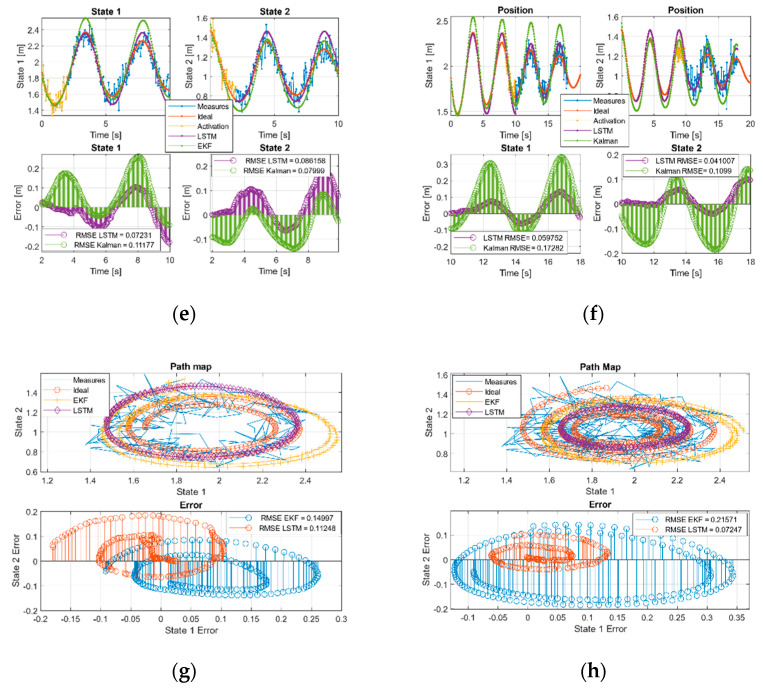
Kalman and LSTM with new feed measurements. (**a**) First and (**b**) second time window URM path evolution. (**c**) First and (**d**) second time window sinusoidal path evolution. (**e**) First and (**f**) second, time window Volterra path evolution in both two states. (**g**) First and (**h**) second window, Volterra phase diagram evolution.

**Figure 4:**
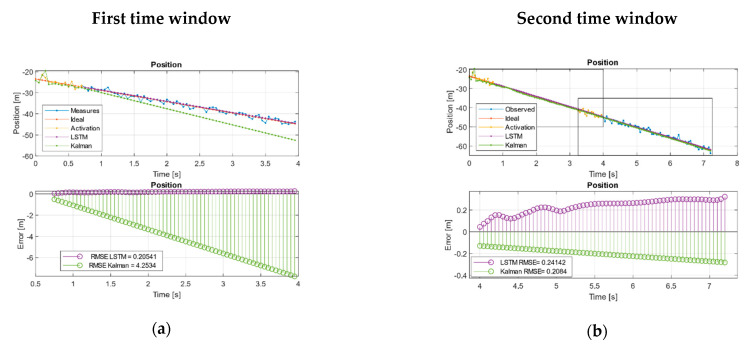

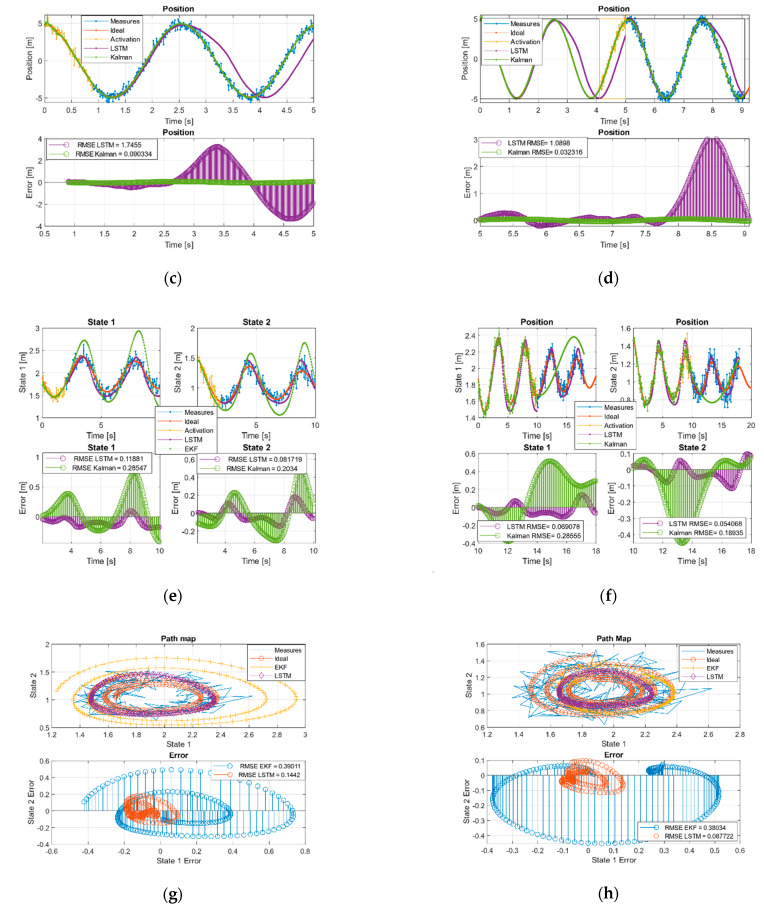
Kalman and LSTM without feed new measurements. (**a**) First and (**b**) second time window URM path evolution. (**c**) First and (**d**) second, time window sinusoidal path evolution. (**e**) First and (**f**) second, time window Volterra path evolution in both states. (**g**) First and (**h**) second window, Volterra phase diagram evolution.

Figure 3a–f and Figure 4a–f show the overlapping regions in yellow—that is, the region without estimates, and is used to activate the networks and also to adjust the KF states in iterative way. After this time, the different systems were fed with new measurements to perform the filtering. In the linear case, this was checked as during the first two time windows, while the KF tended to reduce the RMSE, the network kept the error bounded to acquire the desired trend, Table 7.

In the sinusoidal case, we checked during the first two time windows as the KF tends to reduce its error. In the case of the neural network, it does not manage to improve on the Kalman results, but it remained with an acceptable trend and a comparable RMSE, Table 7.

In the case of Volterra’s system, the trajectory was split into the components defined by the system states. During the first time window, the EKF and the network acquired the system trend but with higher amplitude offset by the EKF than the LSTM, showing a behavior with less error than EKF in the initial moments but with a comparable RMSE. This effect is better observed in Figure 3g (phase diagram first window) where it is shown that, even maintaining a comparable RMSE, the EKF was much farther than the LSTM from the ideal values. During the second time window Figure 3h the effect was even more pronounced, and, this time, we found that the LSTM had a behavior with less error than the EKF. We can see the joint states error in the error diagram of the second time window Figure 3h, where the error in the evolution of the LSTM is shown compressed around (0,0), clearly more compact and reduced than the EKF and, in this case, an order of magnitude higher than the network.

#### 4.4.4. Effect of Missing Observations in the Input Sequence

We simulated the loss of measurements after the overlap/activation region in two consecutive time windows. In the first window, we only used data from the overlap section for network activation and as feed measurements in the Kalman filters. In the second time section, KF used the set of measurements of the first-time window, while the neuronal model only used the overlapping region for the activation. When measurements are missing, the systems were fed with predictions based on the previous estimates from each system as Algorithm 1 explains.

In the case of the URM system, we see how, with few measurements lost, KF can diverge from the real trajectory, while the network managed to extract the trend of the system and maintain a bounded error Figure 4a. On the other hand, when Kalman was fed with a complete time window, it managed to extract a trend that reduced its error compared to the LSTM in terms of the RMSE. However, it may be the case that this is not sufficient and the system continues to decouple as long as the network keeps its error bounded. Figure 4b shows how the Kalman RMSE was lower than the LSTM but with a slightly increasing error trend indicating that it continues to decouple, while the LSTM remained bounded Table 8.

In the case of the sinusoidal paths, we verified how the well-adjusted KF managed to maintain the trends better than the LSTM during the first two-time windows. We also observed how the network managed to have a behavior like Kalman in the first estimation moments, but it decoupled in the absence of measurements and introduced a certain gap in the estimation.

Finally, in the case of the Volterra system, it can be seen how the EKF in the first and second time windows is much more vulnerable and can diverge from the ideal trajectory with respect to the proposed LSTM solution. This is easily observed in each state graphs in Figure 4g,h, especially in the joint state diagrams in error part, where the error of the LSTM is clearly bounded around (0,0) while the EKF is not. Figure 4g,h shows that the EKF was more vulnerable to decoupling in the absence of measurements compared with the neuronal system as observed in the evolution of systems in terms of the amplitude, phase, and finally higher error. 

Figure 4e,f shows that the EKF was more vulnerable to decoupling in the absence of measurements compared with the neuronal system, as observed in the evolution of systems in terms of the amplitude, phase, and definitely higher error. Figure 4g shows how in the first moments around (1.5,1) the EKF, the network, and the ideal measurements evolved together, while the neuronal network extracted the tendency of the equilibrium point and presented an evolutionary behavior on an invariant set, the EKF began to diverge from the limit cycle decoupling itself from the system and becoming unstable in terms of tendency and comparison with the ideal system.

#### 4.4.5. Impact on Filtering of Measurements Simulated with Different Parameters with Respect to the Design 

To perform these experiments we used an ideal model for training and to configure the KF, but we generated new paths with slight changes in the dynamic simulation model with respect to the ideal model.

This α variation was made over each constant’s parameters $\psi_{i}$ of the ideal model, between 5% and 200% of the ideal value. The variation was made with only one parameter to study their impact without changing the rest of the terms with the initial/ideal model. Finally, the new constant $\psi_{i}^{*}$ is as Equation (40), where i indicates the different constants in the dynamic model and j indicates the variation range.

(40) $$\psi_{i}^{*}=\psi_{i}.\alpha_{j}\;$$

For this test, the mean value, the median, and the mode of the set of RMSE values were determined over 1000 new test paths generated over each modification of the constant parameters. This means that, when making 40 modifications, we finally generated 40,000 new paths per study case.

This test was performed on the sinusoidal case by modifying the system frequency and with the Volterra system for each of the four constant terms (34).

**Figure 5:**
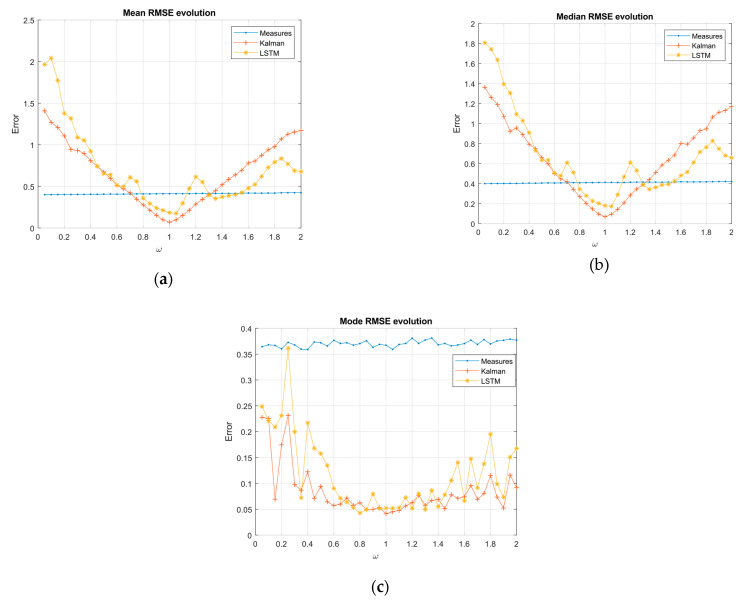
RMSE evolution as the independent term changed in the sinusoidal measurements model: (**a**) RMSE mean, (**b**) RMSE median, and (**c**) RMSE mode.

**Figure 6:**
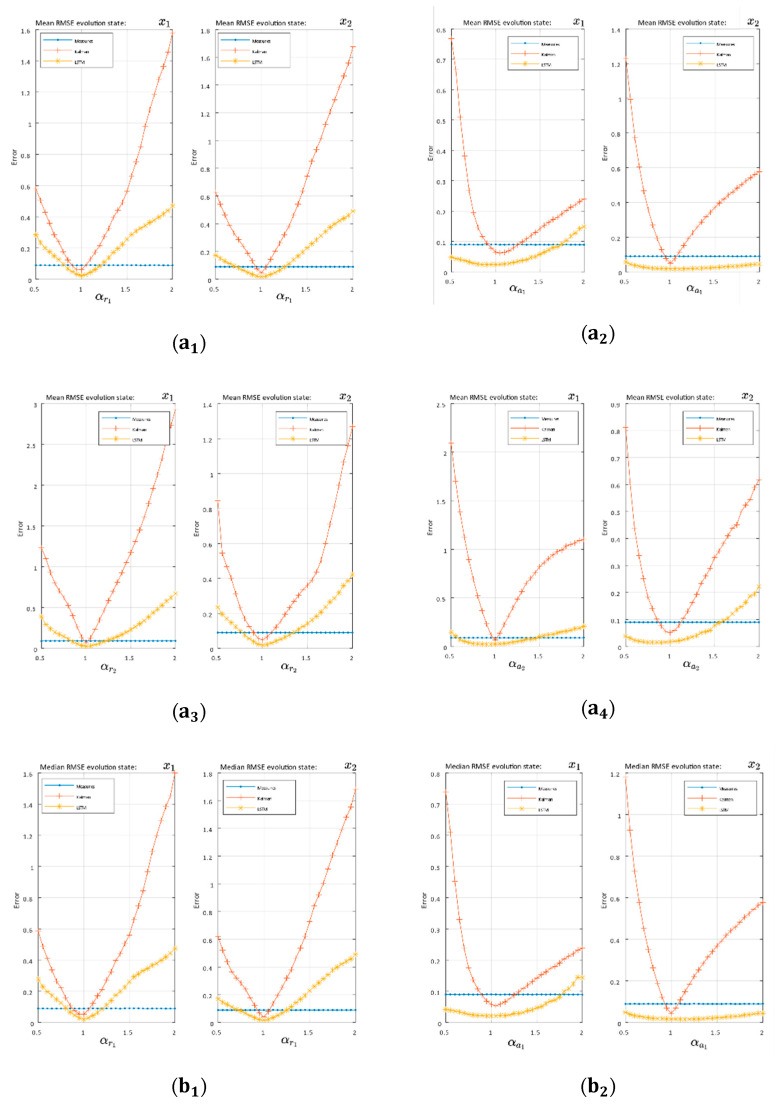

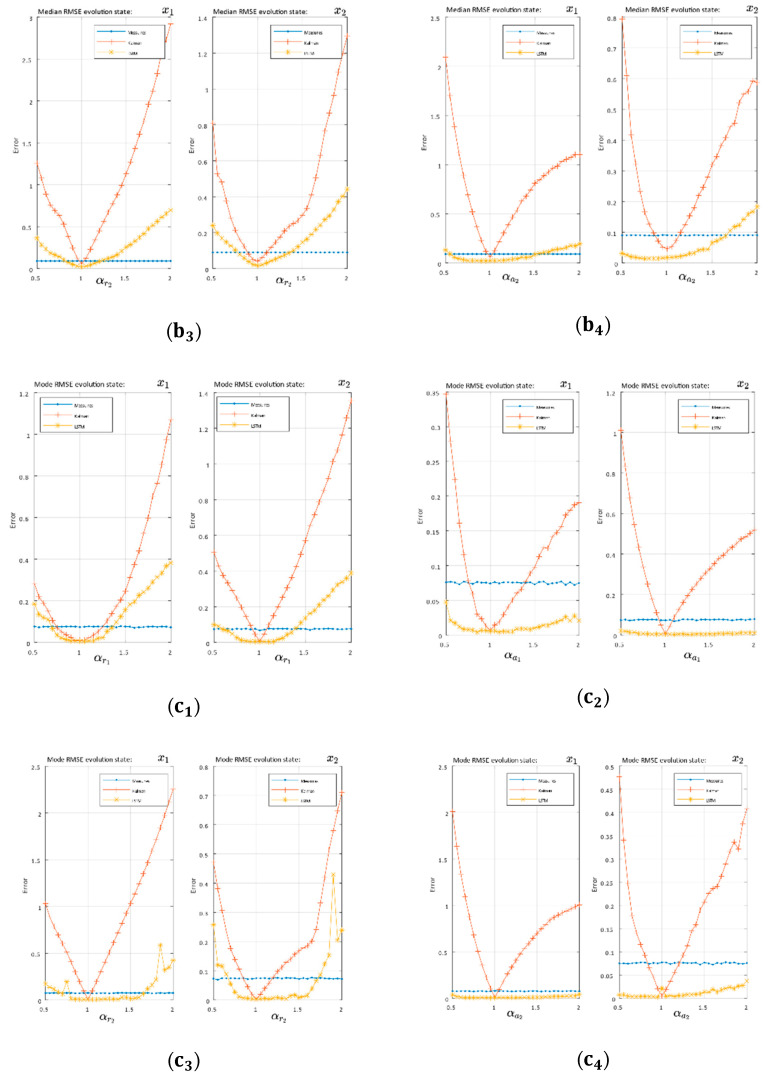
The RMSE evolution as the independent term changed in Volterra model: (**a**) RMSE mean, (**b**) RMSE median, and (**c**) RMSE mode. Subscripts indicate Volterra constant terms: $\left[1,2,3,4]=\;[r_{1},a_{1},r_{2},a_{2}\right]$

In Figure 5 and Figure 6, there were two essential regions in each of the graphs delimited by the variance of the measurements (blue lines). Over this border, the filtering was worse than the measurements; however, this could be due to missing measurements, and so it is interesting to study the evolution over the border of measurements and compare the differences between the classical system and the proposed LSTM system.

Sinusoidal system:

In the sinusoidal case, $\omega^{2}=\psi$ was considered as the constant term. The general RMSE evolution in the average and median KF showed a linear-symmetric growth, while the network showed an irregular behavior, but with an increasing trend on both sides of α = 1. In the lower region of the measure’s variance, Kalman had a lower value than the LSTM, reaching the border after the LSTM in both sides of the optimum. However, we found a region in the range of [1.25, 1.5] in Figure 5a,b in which the network continued filtering while Kalman did not. To the right of this region, Kalman performed worse than the network. In terms of the RMSE frequency (mode), we can see how both systems for the set of ranges studied were maintained in the filtering region and Kalman generally showed the best performance Figure 5c.

Volterra system: 

Based on the statistical values of the mean and median RMSE with Volterra’s system trajectory, the EKF sensitivity to changes in the independent terms are shown in Figure 6a,b. The EKF quickly left the filtering region and showed an increasing trend on both sides of the optimum (α=1). On the other hand, the LSTM architecture was much less sensitive to these changes, becoming practically invariant in the second state $\left(x_{2}\right)$ to $a_{1}$ modifications. The previous trend was generalized for all terms. The mode of the RMSE in Figure 6c showed the same behavior emphasizing the difference between the EKF and the network with the $a_{2}$ constant term modifications, where the network with even a slight increasing trend in the edges did not achieve, in the study range, the filtering border.

## 5. Conclusions

In this work, three neuro-estimator/filters were implemented through a common but different density encoder–decoder architecture, based on recurrent LSTM cells and using the Algorithm 2 design process. These models were compared with a KF adapted to each specific case obtaining similar results in terms of the RMSE but, unlike Kalman, working in only one processing stage. The Kalman algorithm consists of two main processing stages, namely prediction and update, using ad-hoc models, while the proposed solution works in a single stage applying the model built after the training stage.

The study was limited with two consecutive time windows for two linear systems with linear and sinusoidal paths in a one-dimensional path space. In addition, it included a nonlinear autonomous system defined by Volterra–Lotka’s equations, which describes a set of smooth, curved paths in a two-dimensional space. The simulated measurements were made by adding a Gaussian additive term in the state of the system case.

KF has proven to be the optimal process for linear systems; however, the proposed neural architectures, without taking any assumptions as Gaussian, linear, or Markovian processes, managed to show a comparable performance in terms of RMSE Table 5. Although it has been justified why our proposed system does not initially assume Gaussian systems or measurements (Section 3.1), the system has not been tested with other noises to be compared with a reference system, such as KF or EKF. We verified that the system proposed in the case of linear trajectories, with few measurements, managed to acquire the desired trend in front of possible decoupling of the KF in absence of the measurements in Figure 4a,b. When the system had non-linearity, the approaches used in the EKF may diverge from the ideal solution. The neural proposed system managed to improve the behavior of the EKF both in the filtering and in estimation in the absence of measurements Figure 4e–h.

One of the principal advantages of our method lies in the simplicity of modeling the neuro-estimator/filter as KF. Finally, we studied the system behavior in the face of separate trajectories from the models for which the systems had been designed. To do this, we generated new paths modifying each constant term $\psi_{i}$ of the dynamic models by a multiplicative value α. As expected, in all cases, the optimal value was found when the independent term matched between the model and generated values—that is, the multiplicative value α=1.

We proved, as in the case of a linear system (sinusoidal paths), Kalman grew linearly out of the filtering region after the neuronal system. The irregularity of the growth for the neuronal system proposed for sinusoidal paths was shown to exist in regions where Kalman does not work while the network does (understanding by that “work” refers to the filter process).

As far as Volterra’s system is concerned, the influence of each of its four independent terms ($r_{1},a_{1},r_{2},a_{2}$) on EKF systems and the proposed LSTM solution were verified. We checked how the LSTM architecture could be maintained in the filtering area with a higher variation range than Kalman when each one of the independent terms is modified. In the case of $a_{1}$ and $a_{2}$, our system remained practically invariant as shown in Figure 6($a_{2}$,$b_{2}$), Figure 6($a_{4}$,$b_{4}$)-second state $x_{2}$. On the other hand, the EKF with its linear approximations quickly left the filter region in Figure 6. We can affirm that, for all the cases regarding parameter modification on the Volterra system and in the study domain as a whole, the LSTM solution was more robust than the EKF, with the filtering border beyond the EKF or even not having that border in certain cases.

## Figures and Tables

**Table 1:** Synthetic data generation parameters: sinusoidal paths.

Data Generation Range		
Parameter	Minimum	Maximum
$x_{1}$ (m)	−10	10
$x_{2}$ (m/s)	−3	3
**$\omega^{2}\;{\left(rad/s\right)}^{2}$**	6	
Simulation end times (s)	10.01	
Sampling time T (s)	0.01	
Number of window data	500	
Overlap O (Nº data)	90	
**$V\sim N\left(0,\sigma_{Z}\right)$**	0.4	

**Table 2:** Listing of neural network layer with sinusoidal paths: s = 500 samples per input path.

Nr	Name and Type	Activation/Prop.	Learnable	States
1	Sequence Input: 1 × 500	1	-	-
2	lstm_1: LSTM Hidden units: 500	State activation function: tanh Gate activation function: sigm	Input Weights: 2000 × 1 Recurrent Weights: 2000 × 500 Bias: 2000 × 1	Hidden States: 500 × 1 CellState: 500 × 1
3	lstm_2: LSTM Hidden units: 250	State activation function: tanh Gate activation function: sigm	Input Weights: 1000 × 500 Recurrent Weights: 100 × 250 Bias: 1000 × 1	Hidden States: 250 × 1 CellState: 250 × 1
4	lstm_3: LSTM Hidden units: 167	State activation function: tanh Gate activation function: sigm	Input Weights: 668 × 250 Recurrent Weights: 668 × 167 Bias: 668 × 1	Hidden States: 167 × 1 CellState: 167 × 1
5	fc_1: Fully connected	100	Weights: 100 × 167 Bias:100 × 1	-
6	relu_1: ReLU	100	-	-
7	Do: Dropout 20%	100	-	-
8	lstm_4: LSTM Hidden units: 500	State activation function: tanh Gate activation function: sigm	Input Weights: 2000 × 100 RecurrentWeights: 2000 × 500 Bias: 2000 × 1	Hidden States: 500 × 1 CellState: 500 × 1
9	fc_2: Fully connected	1	Weights: 1 × 500 Bias: 1 × 1	-
10	Regression output	Loss function: HMSE	-	-

**Table 3:** Synthetic data generation parameters: Volterra–Lotka paths.

Data Generation Range		
Parameter	Minimum	Maximum
State $x_{1}$	0.8r2a2	1.2r2a2
State $x_{2}$	0.8r1a1	1.2r1a1
**$r_{1},\;r_{2},a_{1}$**	1	
**$a_{2}$**	2	
Simulation end times (s)	20.05	
Sampling time T (s)	0.05	
Number of window data	200	
Overlap O (Nº data)	40	
$V\sim N\left(0,\sigma_{Z_{1}}\right)=N\left(0,\sigma_{Z_{2}}\right)$	0.09	

**Table 4:** Listing of neural network layer: s = 200 is the number of samples per input path.

Nr	Name and Type	Activation/Prop.	Learnable	States
1	Sequence Input: 2 × 200	2	-	-
2	lstm_1: LSTM Hidden units: 400	State activation function: tanh Gate activation function: sigm	Input Weights: 1600 × 2 Recurrent Weights: 1600 × 400 Bias: 1600 × 1	Hidden States: 400 × 1 CellState: 400 × 1
3	fc_1: Fully connected	16	Weights: 16 × 400 Bias: 16 × 1	-
4	relu_1: ReLU	16	-	-
5	Do: Dropout 20%	16	-	-
6	lstm_2: LSTM Hidden units: 200	State activation function: tanh Gate activation function: sigm	Input Weights: 800 × 16 RecurrentWeights: 800 × 200 Bias: 800 × 1	Hidden States: 200 × 1 CellState: 200 × 1
7	fc_2: Fully connected	2	Weights: 2 × 200 Bias: 2 × 1	-
8	Regression output	Loss function: HMSE	-	-

**Table 5:** Kalman and LSTM validation results.

Path-Model	Histogram RMSE $\left[10^{-1}\right]$ (Measurements|Kalman|LSTM)	
	First Checkpoint	Last Checkpoint
Lineal	9.086|4.569|1.444	8.697|2.038|5.799
Sinusoidal	3.971|0.720|1.395	3.955|1.092|1.068
Volterra state $x_{1}$	0.893|0.195|0.424	0.933|0.948|0.089
Volterra state $x_{2}$	0.847|0.168|0.107	0.885|0.501|0.125
Volterra paths (distance)	1.231|0.258|0.437	1.286|1.072|0.153

**Table 6:** The initial simulation conditions.

System-Model	$\mathrm{Initial}\;\mathrm{Conditions}\;{\bar{x}}_{0}$
URM	−23.4897,−5.3815
Sinusoidal	4.8647,−0.9199
Volterra–Lotka	3.0298, 0.8219

**Table 7:** The root mean squared error (RMSE) with continuous feed measurements.

Model	$\mathrm{RMSE}\;\left[10^{-1}\right]\;(\mathrm{Kalman}\;|\;\mathrm{LSTM})$	
	1st Window	2nd Window
Lineal	5.533|1.660	0.969|2.046
Sinusoidal	1.325|1.444	0.678|1.269
Volterra state $x_{1}$	1.118|0.723	1.728|0.597
Volterra state $x_{2}$	0.800|0.861	1.099|0.410
Volterra paths (distance)	1.500|1.125	2.157|0.725

**Table 8:** The RMSE with measurement loss simulation.

Model	$\mathrm{RMSE}\;\left[10^{-1}\right]\;(\mathrm{Kalman}|\mathrm{LSTM})$	
	1st Window	2nd Window
Lineal	42.534|2.054	2.084|2.414
Sinusoidal	0.903|17.455	0.323|10.898
Volterra state $x_{1}$	2.855|1.188	2.855|0.690
Volterra state $x_{2}$	2.034|0.817	1.893|0.541
Volterra paths (distance)	3.901|1.442	3.803|0.877
